# Knowledge, attitudes, and decision making towards prenatal testing among antenatal clinic attendees in Lagos University Teaching Hospital: an institution-based cross-sectional study

**DOI:** 10.11604/pamj.2021.39.106.23667

**Published:** 2021-06-04

**Authors:** Chibuzor Franklin Ogamba, Ochuwa Adiketu Babah, Alero Ann Roberts, Jamaji C Nwanaji-Enwerem, Pamaji Nwanaji-Enwerem, Chibuikem Anthony Ikwuegbuenyi, Oluwaseun Joseph Ologunja

**Affiliations:** 1 Faculty of Clinical Sciences, College of Medicine University of Lagos/Lagos University Teaching Hospital, Lagos, Nigeria,; 2 Department of Obstetrics and Gynaecology, Faculty of Clinical Sciences, College of Medicine University of Lagos/Lagos University Teaching Hospital, Lagos, Nigeria,; 3 Department of Community Medicine and Primary Care, College of Medicine University of Lagos, Lagos, Nigeria,; 4 MD-PhD Program, Harvard Medical School, Boston, Massachusetts, United States of America,; 5 Department of Business and Entrepreneurship, Barber-Scotia College, Concord, North Carolina, United States of America

**Keywords:** Prenatal diagnosis, pregnant women, genetic diseases, congenital anomalies, termination of pregnancy

## Abstract

**Introduction:**

in Africa, genetic diseases and congenital anomalies remain a significant source of morbidity and mortality. Existing data suggests a gap in the use of prenatal tests among pregnant women to better inform decision making. We examined relationships of socio-demographic factors with willingness to terminate affected pregnancies, and the use of, knowledge of, and attitudes towards prenatal screening/diagnostic tests.

**Methods:**

this was a cross-sectional descriptive study of pregnant women who attended antenatal clinics at the Lagos University Teaching Hospital (N = 422) selected by convenience sampling. Responses were obtained with assisted self-administered structured questionnaires.

**Results:**

mean ± S.D. age of the respondents was 32.5 ± 5.3 years. The majority of the participants (92.2%) had at least a secondary education. Ultrasound scans in the second trimester were the most frequently used test (39.1%). Only 77 (18.2%) of the respondents indicated willingness to terminate affected pregnancies. The majority of the respondents had fair knowledge and good attitude scores. Knowledge and attitude scores were significantly correlated (r = 0.25, p < 0.001). Compared to married women, being single was associated with a 2.62-point lower knowledge score (95% CI: -4.63, -0.62, p = 0.01). Compared to women who responded “no” when asked if they were willing to terminate an affected pregnancy, women who responded “maybe” had a 0.81-point lower attitude score (95% CI: -1.45, -0.17, p = 0.01).

**Conclusion:**

our results suggest important socio-demographic differences in women´s knowledge/ behaviours towards prenatal diagnostic tests. Further research is needed to explore these relationships and broader pregnancy-related ethical beliefs among pregnant women in Lagos.

## Introduction

Genetic diseases and birth defects constitute a significant cause of infant morbidity and mortality with about 7.9 million children born with a serious birth defect annually worldwide [1, 2]. Sickle cell disease appears to be the most common genetic disorder in Nigeria with up to a quarter of the population having the sickle cell trait and the disease occurring in about 2%-3% of new-borns-making Nigeria one of the countries with the highest burden of this condition [3]. Chromosomal anomalies like Down syndrome and congenital malformations have also been reported in Nigeria and account for a significant proportion of childhood morbidity and mortality [3]. Nevertheless, there are few comprehensive studies that fully ascertain the prevalence of these conditions. Factors such as a lack of a national new-born screening program, paucities in citizen awareness, and insufficient numbers of diagnostic facilities also contribute to reduced diagnosis rates [3]. The knowledge gained by prenatal diagnostic tests has not only been reported to contribute to the reduction in the incidence of genetic diseases via termination of affected pregnancy, but may also prevent fatal and unfavourable outcomes for the foetus and/or the mother [4, 5]. Advances in genetics research have made prenatal diagnostic tests commercially available. These tests also vary in their forms ranging from non-invasive tests like ultrasound scans, invasive procedures for sample collection such as amniocentesis and chorionic villus sampling, to newer non-invasive prenatal testing (NIPT) using circulating cell-free foetal DNA in maternal blood [5, 6]. Given risks, ethical issues, and moral considerations associated with these tests (e.g. termination of affected pregnancies), especially in Africa, having adequate information about these tests better positions pregnant women in making informed choices about available reproductive options.

With advances in foetal medicine, prenatal diagnosis should increase the possibility of prenatally managing certain congenital anomalies and in resource poor settings like ours, assist health care workers and obstetricians in deciding the best delivery options [7]. Though it has been shown that interventions to improve the quality of prenatal care significantly reduces prenatal deaths, in Nigeria uptake of prenatal diagnostic services are still far from optimum, largely because they remain unavailable to a majority of women as they have not yet been fully incorporated into the routine antenatal screening done in many antenatal clinics [3, 7-9]. The restrictive abortion laws present in the country as well as other ethical challenges hinder legal access to safe termination of affected pregnancies. Studies have been done in this region to ascertain prevalence and reasons for termination of pregnancies among women and have reported prevalence rates; however, the reasons for termination of pregnancies have ranged from fear of school disruption, to young age of last child, to economic challenges associated with being single or having completed family size [10-12]. However, there is a paucity of research investigating the willingness of pregnant women to terminate affected pregnancies after getting a prenatal diagnosis of a congenital anomaly in this region. A recent study carried out among pregnant women attending antenatal clinics in secondary and primary healthcare facilities in Lagos reported a poor knowledge of genetic diseases and prenatal testing, poor attitude scores and a poor use of tests with a majority of the respondents willing to undergo testing but only a minority of the pregnant women expressing willingness to terminate affected pregnancies [13]. This study therefore investigates the relationship between socio-demographic factors, knowledge of and attitudes towards prenatal screening and diagnostic tests, reasons for uptake and non-uptake as well as willingness to terminate affected pregnancies among pregnant women attending antenatal clinics at Lagos University Teaching Hospital Idi-araba Lagos State (a tertiary healthcare centre).

## Methods

***Study design:*** this was a hospital-based cross-sectional descriptive study.

***Study location:*** this study was carried out at the Lagos University Teaching Hospital (LUTH), Idi-araba, Lagos. Lagos State is in the South Western part of Nigeria. LUTH is run by the Federal Government and is one of only two tertiary centers in the state, which serve as referral centers for obstetrics and gynaecology cases in the region.

***Study participants:*** a total of 422 pregnant women who attended antenatal clinics between February 2018 and October 2019 at LUTH participated in this study. Consenting pregnant women were enlisted using a convenience sampling technique. Consenting respondents were recruited as they presented at the hospital for routine antenatal clinics. Pregnant women who were not previously registered and found at the antenatal clinics in LUTH and all those who refused informed consent were excluded from the study.

***Sample size calculation:*** a minimum sample size of 422 was arrived at using the Cochran formula, which gives this study a power of 80%, at a significance level of 95%, and an alpha level of 0.05. An attrition value of 10% was allowed for contingencies such as non-response or recording error.

***Data collection instrument:*** data was collected using an assisted self-administered questionnaire which included both closed and open-ended questions divided into sections. Before it was utilized in the study, the questionnaire was pretested on ten women who attended the antenatal clinic at LUTH at an earlier time. These subjects were included in the final data analysis.

Section A of the questionnaire contained questions examining the socio-demographic characteristics of respondents including age, marital status, religion, highest level of education, employment status, and gravidity. Section B of the questionnaire consisted of sixteen items which determined respondents´ knowledge of prenatal diagnoses, the various prenatal screening and diagnostic tests, as well as an item assessing their major sources of knowledge about prenatal diagnoses. An item asking respondents to indicate which prenatal screening or diagnostic tests they had heard of was also included. Section C of the questionnaire consisted of nine items to test the respondents´ perception of the need for, use, and personal disposition towards prenatal screening and diagnostic tests. It also assessed whether respondents would opt for a diagnostic test if screening tests came out positive or indicated an increased risk. Section D of the questionnaire consisted of two items which assessed respondents´ use of tests. Respondents were also asked to indicate which tests they had made use of and to indicate possible reasons for uptake or non-uptake of the prenatal screening and diagnostic tests listed. Section E of the questionnaire assessed if the respondent would opt to terminate the pregnancy if diagnostic tests came out positive for a genetic disease or congenital anomaly. The respondents were required to indicate ‘Yes’, ‘No’, or ‘I don´t know’. For the knowledge questions, each correct answer was scored as 1 point and each wrong answer was given a score of zero. The maximum attainable score was 16 points and knowledge of the respondents was categorised into three levels: poor (0-5), fair (6-10) and good (11-16). Nevertheless, to maximize statistical power, knowledge score was used as a continuous variable in all analyses. Responses to attitudes were graded on a Likert scale with positive answers given a score of three, negative answers a score of one and respondents who answered “Don´t know” given a score of 2. Respondents who scored eighteen and below were said to have poor attitudes to testing while respondents who scored nineteen and above were said to have good attitudes. Again, attitude score was a continuous variable in the analyses.

### Data analysis

Data was entered and coded using Microsoft Excel 2007 and analysed using R Version 3.6.3 (R Core Team, Vienna, Austria). Descriptive statistics were used to describe the socio-demographic characteristics and the results were presented as means, frequencies and percentages. We first performed Spearman correlations between all continuous variables examined in the analysis (i.e. age, attitude score, and knowledge score). We next employed multi-factor ANOVA models to examine the independence of socio-demographic factors from attitude and knowledge score respectively. Relationships of factors that were statistically significant in ANOVA analyses were further examined using linear regression models adjusted for all socio-demographic variables. Lastly, we performed chi-squared independence tests to examine the independence of categorical socio-demographic factors from willingness to terminate pregnancy - a categorical variable characterized as ‘yes’, ‘no,’; or ‘maybe’. A p-value less than 0.05 was considered statistically significant in all analyses.

***Ethical statement:*** approval for this study was obtained from the health research and ethics committee of the Lagos University Teaching Hospital (LUTH HREC No.: ADM/DCST/HREC/APP/1970). The participants were informed about the significance of the study and how honest and fair answers were important when answering the questionnaires. Consent was sought before administration of the questionnaires. No names were printed on the questionnaires and the respondents were assured of the confidential nature of the study. They were also given the choice to participate or not participate in the study. Filling the questionnaire was subsequently taken as proxy for informed consent.

## Results

### Socio-demographic characteristics of the respondents

Table 1 lists the descriptive characteristics of the study sample. Four hundred and twenty-two (422) pregnant women participated in the study and the response rate was 100%. The mean ± S.D. age of the respondents was 32.5 ± 5.3 years. They were predominantly Christians 359 (85.1%) and married 408 (96.7%). The education level of most of the respondents (92.2%) was at least secondary education and a majority (76.1%) were employed. Most of the respondents (67.5%) were multigravidas.

**Table 1:** study sample characteristics (N=422)

Characteristic	Mean ± SD [Range]	Frequency (%)
Age	32.5 ± 5.3 [18 - 69]	-
Attitude Score	22.5 ± 2.8 [0 - 27]	-
Knowledge Score	7.6 ± 3.2 [ 0 - 16]	-
Education Level		
None	-	7 (1.7)
Primary	-	2 (0.5)
Secondary and Above	-	389 (92.2)
Vocational	-	24 (5.7)
Employment Status		
Employed	-	321 (76.1)
Unemployed	-	101 (23.9)
Marital Status		
Single	-	10 (2.4)
Married	-	408 (96.7)
Separated/Widowed	-	4 (0.9)
Gravidity		
Primigravida	-	137 (32.5)
2 - 4	-	255 (60.4)
More than 4	-	30 (7.1)
Religion		
Christian	-	359 (85.1)
Islam	-	58 (13.7)
Traditional/Other	-	5 (1.2)

### Respondents´ knowledge of prenatal diagnosis

**Figure 1:**
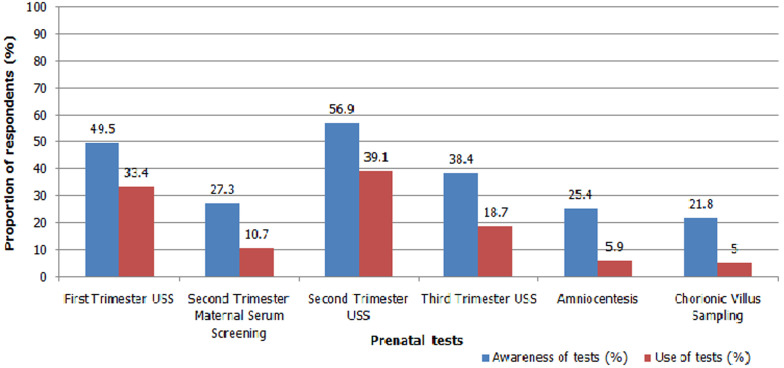
respondents´ awareness of and use of tests; bar chart showing the distribution of respondents who indicated awareness of and use of the various tests listed in study questionnaire

Table 2 further describes the results of the knowledge of prenatal diagnosis survey. The majority of the respondents 307 (72.7%) knew that it was possible to find out if their unborn child had a genetic disease or congenital anomaly and 357 (84.6%) knew that an ultrasound scan could be used to check the age, sex, and well-being of the baby. The majority of the participants 346 (82.0%) also knew that first trimester screening included an ultrasound scan and a blood test and 334 (79.1%) knew that further testing could be done if first trimester screening tests showed that their babies were at increased risk. Two hundred and fifty-one (59.5%) participants knew that tests can be done as early as 11-13 weeks or in the first 3 months to identify pregnancies at risk of Down syndrome. Most of the respondents, 335 (79.4%) knew that a second trimester ultrasound scan can be done between 18 and 22 weeks of pregnancy and a lesser majority knew that it is able to detect heart malformations 238 (56.4%). Most of the respondents 264 (62.6%) knew that further tests can be done to clarify a diagnosis if second trimester maternal serum screening showed an increased risk. Most of the respondents 311 (73.7%) also knew that ultrasound scans can be done in the first, second and third trimesters to detect possibilities of birth defects in the foetus. Over half of the respondents 228 (54.0%) indicated incorrectly that an ultrasound can be used to detect every kind of birth defect. Only 71 (16.8%) respondents knew that second trimester maternal serum screening does not detect only Down syndrome and only 37 (8.8%) knew that a positive screening result does not yet mean that the foetus definitely has Down syndrome or a neural tube defect. Also, only 68 (16.1%) knew that amniocentesis is not a test of the mother´s blood. Sixty-three respondents (14.9%) knew that amniocentesis does not detect only Down syndrome, 28 (6.6%) knew that a negative result from chorionic villus sampling does not guarantee the absence of all birth defects and/or hereditary conditions, and 126 (29.9) knew that there is a chance of miscarriage associated with chorionic villus sampling and amniocentesis. Overall, only 72 (17.1%) of the respondents had good knowledge scores (range: 11-16). Most of the respondents (251, 59.5%) had fair knowledge scores (range: 6-10), and (99, 23.5%) had poor knowledge scores (range: 0-5). Mean knowledge score ± S.D. of the population was 7.6 ± 3.2 which is categorized as fair. Respondents listed health personnel (125, 29.6%), the internet (96, 22.7%) and the media (61, 14.5%) as their major sources of knowledge about prenatal screening and diagnostic tests. Figure 1 depicts the awareness of various prenatal screening and diagnostic tests among the respondents. The majority of the respondents were aware of ultrasound scans done in the second trimester to detect foetal anomalies (240, 56.9%) and a little less than half (209, 49.5%) of the respondents were aware of first trimester ultrasound scans. A smaller fraction of the respondents was aware of second trimester maternal serum screening for Down syndrome and neural tube defects (115, 27.3%), third trimester ultrasound scan for urinary tract abnormalities (162, 38.4%), diagnostic tests such as amniocentesis (107, 25.4%), and chorionic villus sampling (92, 21.8%).

**Table 2:** knowledge of prenatal diagnosis survey

Characteristics	Yes	No	Don't know
**Knowledge of Prenatal diagnosis**			
It is possible to find out if my unborn child has a genetic disease or birth defect.	307 (72.7)	24 (5.7)	91 (21.6)
An ultrasound can be used to detect every kind of birth defect	228 (54.0)	92 (21.8)	102 (24.2)
The main use of an ultrasound is to check the age, sex and well-being of the baby.	357 (84.6)	33 (7.8)	32 (7.6)
First trimester screening involves ultrasound and a maternal blood test.	346 (82.0)	8 (1.9)	68 (16.1)
Further tests can be done to clarify a diagnosis if a first trimester screening test shows at increased risk.	334 (79.1)	9 (2.1)	79 (18.7)
Tests can be done as early as 11-13 weeks or in the first 3 months to identify pregnancies at risk of Down syndrome	251 (59.5)	25 (5.9)	146 (34.6)
Second trimester ultrasound scan can be done between 18 and 22 weeks of pregnancy.	335 (79.4)	12 (2.8)	75 (17.8)
Second trimester ultrasound scan at 22 weeks detects heart malformations	238 (56.4)	11 (2.6)	173 (41.0)
Second trimester maternal serum screening detects only Down syndrome	65 (15.4)	71 (16.8)	286 (67.7)
Further tests can be done to clarify a diagnosis if second trimester maternal serum screening shows at increased risk.	264 (62.6)	3 (0.7)	155 (36.7)
A positive screening result means that the foetus definitely has Down syndrome or a neural tube defect.	149 (35.3)	37 (8.8)	236 (55.9)
Ultrasound scans can be done in the first, second and trimesters to detect possibilities of birth defects in the foetus.	311 (73.7)	14 (3.3)	97 (23.0)
Amniocentesis is a test of the mother´s blood.	86 (20.4)	68 (16.1)	268 (63.5)
Amniocentesis is a test that detects only Down syndrome.	51 (12.1)	63 (14.9)	308 (73.0)
A negative result from a chorionic villus sampling guarantees the absence of all birth defects and/or hereditary conditions	90 (21.3)	28 (6.6)	304 (72.0)
There is a chance of miscarriage associated with chorionic villus sampling and amniocentesis.	126 (29.9)	9 (2.1)	287 (68.0)

### Respondents´ attitudes to prenatal diagnosis

A majority of the respondents (352, 83.4%) felt that prenatal screening tests were valuable, 343 (81.3%) felt that they should be offered to all pregnant mothers, 314 (74.4%) that prenatal diagnosis will help doctors find a cure for genetic diseases and birth defects in children, 279 (66.1%) that the cost of prenatal diagnosis should not influence whether they are done or not, and 348 (82.5%) that they could plan for the future with information gained from prenatal diagnosis. Also, a majority of the respondents (338, 80.1%) felt that prenatal screening and diagnosis would help reduce anxiety during their pregnancy and 317 (75.1%) would opt for a diagnostic test to further confirm the results, if prenatal screening tests showed there was a cause for concern. Only 140 (33.2%) respondents felt they would accept the accuracy of tests, and a majority (296, 70.1%) would worry about how these tests would affect the health of their baby. Overall, a majority of the respondents (378, 89.6%) had good attitude scores. The mean ± SD of attitude scores was 22.5 ± 2.8 which is characterized as good. Attitude and knowledge scores were significantly correlated (r = 0.25, p < 0.001) with each other, but neither were correlated with chronological age.

### Respondents´ use of tests

Figure 1 also shows the use of various tests by the respondents. Ultrasound scans in the second trimester were the most frequently used test (165, 39.1%), followed by first trimester ultrasound scans and blood tests (141, 33.4%), third trimester ultrasound scan (79, 18.7%), second trimester maternal serum screening (45, 10.7%), amniocentesis (25, 5.9%), and chorionic villus sampling (21, 5.0%).

***Reasons for uptake of prenatal diagnostic tests:*** among the reasons given for uptake of prenatal diagnostic tests, the major reason indicated was to make sure the baby was healthy 179 (42.4%). Other notable reasons included to see the baby 141 (33.4%), that the tests were routinely done when they are pregnant 109 (25.8%), conformity with the care provider´s suggestion 96 (22.7%), that they were concerned about the risk of birth defects and/or hereditary conditions being passed on to their children 77 (18.2%), that there was a positive family history of a hereditary condition 21(5.0%), that they were aged 35 to 37 years or older, 49 (11.6%), that they have had more than two miscarriages 22 (5.2%), and that they are closely related to their partners 22 (5.2%).

***Reasons for non-uptake of prenatal diagnostic tests:*** among the reasons given for non-uptake of prenatal diagnostic tests, the major reason (98, 23.2%) respondents indicated was that they did not know about them, 41 (9.7%) felt that they were not necessary, 20 (4.7%) since they were not aged 35 to 37 years or older, 17 (4.0%) respondents reported that they could not afford it. Less commonly, 12 (2.8%) respondents reported that they were not concerned about the risk of genetic diseases or hereditary conditions, 9 (2.1%) said that the tests were not available, 5 (1.2%) respondents did not want to be faced with a difficult decision, 3 (0.7%) felt that the risk was too high, and one respondent (0.2%) indicated that she had not been asked to do the tests.

### Respondents´ willingness to terminate affected pregnancies

Only 77 (18.2%) of the respondents indicated that they were willing to terminate affected pregnancies, 100 (23.7%) indicated that they were not willing to terminate affected pregnancies, and a majority of the population 245 (58.1%) was indecisive on the subject. Chi-squared independence tests did not reveal any statistically significant relationships of categorical variables with willingness to terminate pregnancy.

### Relationships of socio-demographic factors with attitude and knowledge scores

Multifactor ANOVA of socio-demographic factors with attitude score demonstrated statistically significant relationships of education (p = 0.04), gravidity (p = 0.04), and decision to terminate with attitude score (p = 0.003). Subsequent linear regression models with all socio-demographic variables as covariates also demonstrated statistically significant relations of gravidity and willingness to terminate pregnancy with attitude scores. Specifically, compared to women with a gravidity of 1, on average women with a gravidity of 2 had a 1.04-point lower attitude score (p = 0.002). However, no other relationships between gravidity groups reached statistical significance. In the same model, compared to women not willing to terminate their pregnancies, women who answered maybe on average had a 0.81-point lower attitude score (p = 0.01, Table 3). Again, no other factors had statistically significant relationships with willingness to terminate. No demographic factors demonstrated statistically significant relationships with knowledge score in the multifactor ANOVA model. However, in the joint linear regression model, compared to women who were married, single women had a 2.62-point lower knowledge score (p = 0.01, Table 3).

**Table 3:** socio-demographic factors as joint predictors of prenatal diagnosis knowledge and attitude scores (N = 422)

Variables	Change in Knowledge Score (95% CI)	P-value	Change in Attitude Score (95% CI)	P-value
**Age**	-0.06 (-0.12, 0.01)	0.10	-0.003 (-0.06, 0.06)	0.92
**Education**				
Primary	-3.74 (-8.71, 1.23)	0.14	-1.64 (-5.98, 2.68)	0.46
Secondary and Above	0.26 (-2.17, 2.69)	0.83	0.81 (-1.31, 2.93)	0.45
Vocational	-0.81 (-3.53, 1.91)	0.56	-0.36 (-2.72, 2.01)	0.77
**Employment**				
Unemployed	ref	-	ref	-
Employed	0.47 (-0.25, 1.19)	0.20	0.25 (-0.38, 0.87)	0.44
**Marital Status**				
Married	ref	-	ref	-
Single	-2.62 (-4.63, -0.62)	*0.01	0.86 (-0.88, 2.61)	0.33
Separated	0.83 (-2.77, 4.43)	0.65	0.75 (-2.39, 3.88)	0.64
Widow	3.68 (-2.94, 10.31)	0.28	-2.20 (-7.98, 3.57)	0.45
**Gravidity**				
1	ref	-	ref	-
2	-0.56 (-1.32, 0.20)	0.15	-1.04 (-1.71, -0.39)	*0.002
3	-0.63 (-1.57, 0.30)	0.18	-0.31 (-1.12, 0.51)	0.46
4	0.42 (-0.64, 1.48)	0.44	-0.40 (-1.32, 0.53)	0.40
>4	0.24 (-1.10, 1.57)	0.73	-0.20 (-1.36, 0.97)	0.74
**Religion**				
Christian	ref	-	ref	-
Islam	-0.49 (-1.36, 0.39)	0.28	0.06 (-0.71, 0.82)	0.88
Other	-1.07 (-3.86, 1.71)	0.45	0.91 (-1.51, 3.34)	0.46
**Willingness to Terminate**				
No	ref	-	ref	-
Maybe	-0.56 (-1.30, 0.17)	0.13	-0.81 (-1.45, -0.17)	*0.01
Yes	0.10 (-0.84, 1.05)	0.83	0.27 (-0.55, 1.10)	0.52

* statistically significant values

## Discussion

In this study of women attending antenatal clinics at LUTH, we describe relationships of socio-demographic factors with knowledge of prenatal diagnostic tests, attitude scores, and willingness to terminate affected pregnancies. Most of the women in this study were in their fourth decade of life. Previous studies have reported an increased incidence of major congenital anomalies in this age group [14, 15]. Therefore, we hypothesized that - on average - women in this population should be well informed about such a possibility and various modalities for screening and diagnosis. We also hypothesized that pregnant women in this age group would have some awareness of the various ethical issues associated with these conditions so that they will be able to make informed choices as regards the fate and health of their unborn children. Health personnel, the internet and the media were listed as major sources of knowledge by the respondents. This goes to show that health professionals have a significant impact on pregnant women with regards to creating awareness and improving their knowledge of prenatal diagnosis. However, the fact that only a small proportion (29%) of the women reported getting information on prenatal screening and diagnostic testing from the health personnel signifies lapses in health education and counselling in our health institution. Mean knowledge score of the respondents was 47.5% (7.6/16). The mean knowledge score was also slightly lower than observed in a similar study in in Western Australia (62%) [16], but higher than in a similar study done in primary and secondary health facilities in Lagos (21%) [13]. This finding suggests that populations receiving care from tertiary centres, like our own study sample, receive greater health education than populations receiving care in lower resourced health facilities. Furthermore, tertiary health institutions often have better research facilities and health personnel with higher training/qualifications. Still, the precise reasons for these differences merit further investigation.

Although populations receiving care at tertiary centres like LUTH seem to be more knowledgeable about their health, our study still highlights important deficits in their health literacy. A majority of the respondents did not know about prenatal diagnostic tests like amniocentesis and chorionic villus sampling, which is contrary to the findings of a similar study done in Northern Italy where these prenatal invasive tests were known by 58.7% of the women [17]. Furthermore, a majority of the respondents felt that an ultrasound could detect every birth defect. The major reason given for non-uptake of these tests (23.2%) was a lack of knowledge about them. This can be largely due to the unavailability of these diagnostic facilities and skills in low resource countries like ours. There is therefore a need to further develop facilities for foetal medicine in Nigeria and further train Obstetricians in this sub-specialty. Most of the respondents in our study population had good attitudes to prenatal diagnosis. Some of the respondents were, however, worried about the cost of tests, accuracy of test results and safety of diagnostic tests in pregnancy. Good attitude scores were also reported in Ibadan and in Romania [4, 18]. Our ANOVA models demonstrated relationships of attitude scores with education in our study sample. Attitude scores were correlated with education in the study in Ibadan [4]. Nonetheless, these relationships were null in our multivariate linear models. Attitudes, however, did demonstrate relationships in *secundigravidas* and women who were unsure about termination of pregnancy. We hypothesize that women who are unsure about their willingness to terminate pregnancies would have a less than positive attitude about prenatal diagnoses, however this remains unclear - and merits further evaluation. Despite the degree of awareness of screening tests in our population and the fact that a good number of the respondents were experiencing their second or higher pregnancy, only a minority indicated that they had made use of the categories of screening tests listed in our study, contrary to the findings of a study done in the Netherlands where uptake rates for foetal anomaly scans were high [19]. This suggests a need for more communication between physicians and patients, which may be a challenge considering the peculiarities of healthcare practice in our setting.

A minority of the respondents indicated that they were willing to terminate pregnancies, a slightly higher proportion than the willing group were not open to the idea. Majority of the respondents were unsure about this option. This is also similar to the findings of a previous study in Lagos, where a minority (10.1%) of the respondents expressed willingness to terminate affected pregnancies [13]. We found no significant correlates of willingness to terminate affected pregnancies among our socio-demographic factors. Based on this, we hypothesize that many pregnant women in our population are not comfortable making this decision with more women unwilling to terminate affected pregnancies than those who are willing. A recent study conducted in this region among women with abortion complications reported that some women who were on medication when they found out they were pregnant opted to terminate the pregnancy for fear of having a child with congenital anomalies [20]. More studies need to assess reasons and factors associated with decisions for or against termination of pregnancies affected with genetic diseases and congenital anomalies.

Our study possesses several strengths including a moderate sample size, use of a comprehensive survey instrument, and the collection of data related to important socio-demographic factors. Nevertheless, we do have some notable limitations. As a tertiary hospital-based convenience sampling study, findings may not be accurately generalised to most of the population. Also, some of the significant associations may likely be due to chance as there was no adjustment for multiple hypothesis testing. Lastly, we conducted a cross-sectional descriptive study. Hence, our results are limited with respect to the causal direction of reported findings.

## Conclusion

In summary, there is room for improved knowledge, accessibility to and use of prenatal screening and diagnostic tests among pregnant women in Lagos, Nigeria. Ethical issues associated with termination of pregnancies affected with genetic diseases and congenital anomalies need to be explored to better understand the choices of pregnant women in our population with regard to the fate of their unborn children.

### What is known about this topic

- Genetic diseases and congenital anomalies are common in Nigeria;
- Prenatal diagnostic services for genetic disease and congenital anomalies have not reached optimal standards in Nigeria and termination of pregnancies is legally restricted;
- Knowledge and use of prenatal diagnosis and available reproductive options have been shown to improve pregnancy outcomes and reduce complications in the management of affected pregnancies.

### What this study adds

- This study assessed the knowledge of, attitudes towards and use of prenatal screening/diagnostic tests among pregnant women in Lagos and associated factors;
- This study assessed possible reasons influencing uptake or non-uptake of tests among pregnant women in Lagos;
- This study determined the willingness of pregnant women in Lagos to terminate affected pregnancies based on results of tests.
